# Economy and the Food Question

**Published:** 1915-08-07

**Authors:** Joseph Collinson

**Affiliations:** Animals' Friend Society, York House, Portugal Street.


*To the Editor of* The Hospital.

### Economy and the Food Question.

*To the Editor of* The Hospital.

Sir,—With your permission, I should like to suggest to those of your readers who have given some thought to the economical aspect of the food question that the present is an excellent opportunity to test the value of the meatless diet, for the adoption of which there are various valid reasons. Lest any accustomed to the old order of things should think that the abandonment of butcher's meat would be a terrible leap in the dark, it may be mentioned that Professor Sims Woodhead and Professor James Long, neither of whom is a "convinced" vegetarian, have recently testified to the fact that a well-balanced dietary of vegetable origin is all-sufficient to sustain men in the highest condition both of body and mind. The non-flesh diet has, indeed, everything to recommend it; it is hygienic, economic, and humane, and in such a crisis as we are now passing through it certainly seems worth while for the civilian population to give the matter their consideration.

There are many useful cookery books, published at 1d. and upwards, giving numerous recipes for the preparation of choice meals from eggs, cheese, beans, peas, lentils, macaroni, rice, oatmeal, maize-meal, and potatoes (the two latter being the best and cheapest of all foodstuffs), which may be used at all times in the place of the interminable dishes of beef, mutton, pork, or bacon.—I am, yours faithfully, Joseph Collinson.

Animals' Friend Society, York House,
Portugal Street.

[Cookery books on meatless diet are generally vitiated by being written by faddists, and of all faddists the food faddist is perhaps the worst. One of the best guides to every aspect of the subject written of late years is "Common Sense Dietetics," by C. Louis Leipoldt, F.R.C.S., which is written valiantly up to its title.—Ed. The Hospital.]

